# A scoping review of current approaches to strengths-based transition practices for autistic adolescents

**DOI:** 10.1177/13623613251346336

**Published:** 2025-06-25

**Authors:** Sophie Rumsa, Bahareh Afsharnejad, Elinda Ai Lim Lee, Sven Bölte, Tele Tan, Sonya Girdler

**Affiliations:** 1 Curtin Autism Research Group, Curtin University, Perth, Western Australia; 2 Curtin School of Allied Health, Curtin University, Perth, Western Australia; 3 School of Population Health, Curtin University, Perth, Western Australia; 4 Center of Neurodevelopmental Disorders (KIND), Centre for Psychiatry Research, Division of Neuropsychiatry, Department of Women’s and Children’s Health, Karolinska Institutet, Stockholm, Sweden; 5 Child and Adolescent Psychiatry, Stockholm Health Care Services, Stockholm County Council, Stockholm, Sweden; 6 School of Electrical Engineering, Computing and Mathematical Sciences, Curtin University, Perth, Western Australia; 7 School of Allied Health, University of Western Australia, Perth, Western Australia

**Keywords:** adolescents, adult outcomes, autism spectrum disorders, strengths-based

## Abstract

This review aimed to map the literature surrounding strengths-based approaches in studies with autistic adolescents focusing on adult transition outcomes, by describing the active ingredients of interventions and the properties of assessment tools. Relevant databases were searched for articles surrounding strengths-based practices, which met the following criteria: (a) written in English, (b) published since 1990, and (c) involving adolescent participants with a diagnosis of autism spectrum disorder. Data extraction included sample, aim, intervention description, outcome measures, and results. Data were synthesised using a descriptive approach and thematic analysis was conducted to identify active ingredients, practice strategies, outcomes, and assessment properties. Overall, 35 articles met the inclusion criteria, including 26 studies evaluating strengths-based interventions and 9 articles relating to general transition outcomes. Five types of interventions were classified according to their active components: transition planning, employment preparation and training, technology programmes, peer mentoring, and cognitive skills training. The best practice themes highlighted the importance of leveraging each individual’s interests and skills, developing individualised processes, and accommodating participant’s needs through a holistic approach. Analysis of the outcome measures highlighted a gap in standardised strengths-based assessments, but uncovered primary outcome themes of self-determination, social skills, confidence, and work-related skills.

**Lay abstract:**

This review explores research on strengths-based approaches for autistic adolescents, particularly focusing on outcomes related to transitioning into adulthood. The goal was to identify and describe the key components of strengths-based interventions and assessment tools. The review looked at studies published since 1990 that involved adolescents with a diagnosis of autism spectrum disorder and included a variety of approaches aimed at promoting positive adult outcomes. After analysing 35 relevant articles, the review categorised five types of interventions based on their key components: transition planning, employment preparation, technology programmes, peer mentoring, and cognitive skills training. The review also identified fewer strengths-based measures than expected, with many studies employing custom measures. This points to a gap in reliable strengths assessments for autistic adolescents, which would be useful in transition planning and improving self-image. Despite the lack of standardised tools, the review highlighted several common strengths-based outcomes, such as improvements in self-determination, social skills, confidence, and work-related skills.

## Introduction

Traditionally, interventions for autistic individuals have focused on identifying and addressing the challenges or deficits associated with autism (Guldberg, 2017). These approaches have been largely underpinned by viewing autism solely from a biological perspective of disability, aiming to address autistic symptomatology (Anderson-Chavarria, 2022; Gold, 2014). These interventions largely emphasise the difficulties faced by autistic individuals, including communication barriers, social skill differences, or challenging behaviours (Kapp, 2022). While deficit-based approaches can provide necessary support, they often overlook or minimise the strengths and unique perspectives of autistic individuals (Anderson-Chavarria, 2022). This has led to a growing emphasis on strengths-based (SB) or neurodiversity-affirming approaches, which recognise and build on the inherent strengths and abilities of autistic individuals (Armstrong, 2011).

The neurodiversity paradigm has emerged as a contemporary approach to autism, and other neurodevelopmental conditions, conceptualising these phenomena as neurodivergence stemming from natural variation in human minds (Baron-Cohen, 2017). The term neurodiversity was developed through autistic activist communities in the 1990s who posed the idea that being neurotypical was perhaps its own disorder, if we were to consider the challenges in the same way we do with neurodivergence (Blume, 1998; Botha et al., 2024). The term was further refined by neurodivergent sociologist, Judy Singer, who called for a more ecological view of society, with mutual recognition of different styles of being, and no style being typecast as either right or wrong (Singer, 1999). While advocates acknowledge that autism is, in fact, a disability, some suggest that impairment is the product of a limiting environment, as opposed to limitations of the individual (Anderson-Chavarria, 2022; den Houting, 2019). This is aligned with the social model of disability which prioritises creating a person-environment fit to improve outcomes (Burnham Riosa et al., 2017; Wehman et al., 2017). However, the social model has been criticised for focusing solely on impairments within society and overlooking the significance of individual functionality (Shakespeare & Watson, 2001). The neurodiversity approach is perhaps better defined as recognising the interaction of an individual’s characteristics within their environment (Dwyer, 2022). Interventions underpinned by this framework acknowledge both the skills and abilities of autistic individuals, and the facilitators and barriers present within their environment (Bölte et al., 2021; Shakespeare & Watson, 2001).

The period of adolescence is exemplar of the limiting environment–character interaction, in which increasing social demands often exacerbate the social difficulties associated with autism (Picci & Scherf, 2015). This particularly challenging period often results in poor transition outcomes for autistic youth, such as low employment rates, low job retention, and lower rates of post-secondary qualification completion (Nicholas & Klag, 2020; Taylor & Seltzer, 2011). However, research demonstrates significantly higher success rates when individuals have sufficient support prior to the transition (Hatfield et al., 2017b; Wehman et al., 2014). The evidence suggests that approaches focusing on the strengths of autistic young people enhance their self-esteem and confidence, help them to recognise and develop their skills, and foster a sense of belonging with their peers (Bölte, 2021; Lee et al., 2020). Conversely, failing to recognise individuals’ strengths can contribute to poor educational and employment outcomes (Black et al., 2020; Scott et al., 2018). Furthermore, outcomes such as poor job retention and mental health challenges in autistic adults may, at least in part, be attributed to an insufficient person-environment fit (Bölte, 2021; Pfeiffer et al., 2018). Clearly, acknowledging and leveraging the strengths of school-aged autistic youth is a priority for working towards improving the post-school outcomes for this group.

Common strengths among autistic individuals include enhanced visual-spatial processing, sensory perception, attention, memory, and the ability to understand complex patterns or systems (Baron-Cohen et al., 2009; Wright et al., 2020). These traits can be highly beneficial in the workforce and a practical approach to SB intervention is to explore career opportunities which match the individuals’ strengths and goals (Dean, Hagiwara, et al., 2022; Hatfield, Falkmer, et al., 2018). What’s more, autistic individuals often have a predisposition towards special interest areas, on which adolescents may hyperfocus and gain expertise, contributing to high performance in technology, mathematics, and the arts (de Schipper et al., 2016). Educational and extra-curricular programmes leveraging these interests are also emerging as a beneficial approach in transition preparation (Bross & Travers, 2017). The majority of research has focused on evaluating programmes targeting autistic youth with an interest in science, technology, engineering, arts, and maths (STEAM), building their skills with a view to future employment opportunities (Dunn et al., 2015; Jones et al., 2021).

Despite the increasing popularity and rhetoric surrounding SB approaches, to date there is no consensus as to what exactly defines a SB approach in autism. A recent review concluded that, in school settings, SB approaches for autistic students involve leveraging strengths, developing relationships, and adjusting environmental factors (J. White et al., 2023). While another review found that SB technology programmes with autistic students were formed around three core elements: mutual respect, demonstrating skills, and personal interests (Jones et al., 2022). Taken together, we can start to envisage what it means to employ a SB approach; however, the literature lacks a clear understanding of the core components attributed with producing the desired outcomes, and properties of the assessments utilised to measure strengths and SB outcomes with autistic youth. Given the potential of SB approaches to improve the transition outcomes of autistic youth, clarity surrounding these aspects is crucial to inform the development of future programmes.

The present review aimed to comprehensively map the literature surrounding SB approaches in the context of improving transition outcomes for autistic adolescents. With a broad range of practices anticipated, two research questions were formulated: In the context of autistic adolescents transitioning to adulthood: (1) what are the active ingredients of SB interventions and (2) what are the properties of SB assessment tools?

## Methods

A scoping review was chosen as the most appropriate method due to the broad nature of the research question and need to include research employing a range of research methods. Scoping reviews intend to map the key concepts of a research area, identifying gaps prior to further investigation (Daudt et al., 2013). This approach lends to addressing broader topics where a number of study designs might be applicable to the review (Arksey & O’Malley, 2005). A preliminary search of MEDLINE, the Cochrane Database of Systematic Reviews, and *Joanna Briggs Institute Evidence Synthesis* was conducted which identified no published or in-progress systematic reviews or scoping reviews on the topic. In line with the scoping review methodological framework as outlined by Arksey and O’Malley (2005), and refined by Daudt et al. (2013) and Levac et al. (2010), this review was undertaken in six steps: identifying the research question; identifying relevant studies; study selection; charting the data; collating, summarising, and reporting the results; and consulting with stakeholders.

### Search strategy

The search strategy of a scoping review is broad and comprehensive, utilising multiple sources to find all relevant literature (Levac et al., 2010). For this review, online databases MEDLINE, PsycINFO, CINAHL, and Pro-Quest were searched for articles meeting the following criteria: (a) written in English; (b) published between 1990 and 2022; and (c) involving adolescent participants with a diagnosis of autism spectrum disorder (ASD), as per the *Diagnostic and Statistical Manual of Mental Disorders*, fifth or fourth edition (DSM-5 or DSM-IV; American Psychiatric Association, 2013). To ensure breadth of coverage, the inclusion and exclusion criteria to identify the primary studies were developed post hoc. Key search terms were determined under three groups: (1) diagnosis; (2) age; and (3) focus of the research (Table 1). Combinations of terms between groups were identified, truncated, and exploded to achieve optimal search results.

**Table 1.** Key search terms.

Diagnosis	Age	Focus
autistic or autism or ASD or asperger* or neurodiver*	adolescen* or teen* or youth* or ‘young adult*’ or adulthood	strength* or self-determin* or goal* or skill*

* Search terms truncated and exploded.

### Study selection

Starting with a broad search, an iterative process was followed in which the searches were refined and articles were reviewed until the search terms and inclusion criteria were clarified (Levac et al., 2010). Studies were included if they aligned with the following criteria:

#### Participants

Adolescents aged 12–21 years were included, with the upper limit reflecting access to transition services under American legislation, the Individuals with Disabilities Education Improvement Act (IDEIA, 2004), which supports autistic individuals until they reach age 22. Participants were required to have a diagnosis of ASD, be responding on behalf of an adolescent with a diagnosis of ASD, or be responding about an adolescent with ASD. Diagnosis may have been confirmed through a letter or clinical record, by virtue of enrolment in an autism-specific programme or school, or participant self/proxy-report. No exclusion criteria regarding participants were developed.

#### Concept

The article described a SB transition intervention, or an assessment measure targeting autistic students transitioning out of high school, or an evaluation of stakeholder perspectives surrounding desired transition practices and outcomes. Articles were evaluated for the use of neurodiversity-affirming language reflecting alignment with the SB approach (Bottema-Beutel et al., 2020). Those that included ableist language, such as referring to ‘treatment’ in the context of improving functional skills (e.g. treating sensory challenges to improve independence), were excluded. This criterion ensured the selected articles adhered to both neurodiversity-affirming language and the evidence that interventions focused on functional treatment for autistic individuals may result in the loss of specialised skills (Cherewick & Matergia, 2024).

#### Research design

Both experimental and quasi-experimental study designs, including randomised controlled trials, non-randomised controlled trials, before and after studies, and interrupted time-series studies, were included. In addition, analytical observational studies including prospective and retrospective cohort studies, case-control studies, and analytical cross-sectional studies were considered for inclusion. This review also considered descriptive observational study designs including case series, individual case reports, and descriptive cross-sectional studies for inclusion.

Qualitative studies were also considered, including, but not limited to, designs such as phenomenology, grounded theory, ethnography, and qualitative description. Mixed-method designs incorporating both quantitative and qualitative data were also considered. Educational and clinical guidelines were considered, depending on the ability of the content to answer the research questions. Existing systematic and literature reviews, as well as dissertations, book chapters, and opinion papers, were not considered.

Journal articles originating from any country were considered, but were required to be published in English given the potential loss of qualitative data validity through the translation of quotations (McKenna, 2022; van Nes et al., 2010). Only papers published since 1990 were considered given the significant changes in this research field over time, including diagnosis and intervention approaches (O’Reilly et al., 2020).

Following the search, all identified citations were collated and uploaded into EndNote 20 (The EndNote Team, 2013) and duplicates were removed. Titles were then screened by the primary researcher to delete any highly irrelevant articles, before uploading all remaining articles into the screening software, Research Screener (Chai et al., 2021). The software uses semi-automation to present articles in order of relevance based on five highly relevant seed abstracts initially identified by the researchers. Presenting abstracts likely to be relevant in rounds of 50, the programme actively learns based on which of those are flagged relevant by the reviewer. In the present review, a data-driven stopping criterion was applied, where screening continued until 7% of the sample uploaded to the screener was consecutively classified as irrelevant, thereby satisfying the threshold for stopping (Campos et al., 2024). A secondary researcher screened 10% of the abstracts and the software assisted in the process of identifying conflicts. Through two meetings, the conflicts were discussed and resolved by the first two authors and updated in Research Screener. The articles were then exported for full-text review by the first author.

### Charting the data

A framework for charting the data of intervention-based studies was developed, depending on the type of study: qualitative, quantitative, or mixed method (Arksey & O’Malley, 2005). Characteristics of the studies were charted using the following headings: year, location, aim, sample, methodology, duration, intervention type (IT), outcome measures (quantitative and mixed-method studies), results, and quality analysis (Supplementary material Table 1). Studies relating to transition outcomes excluding those evaluating an intervention were charted using the following headings: year, location, sample, aim, methodology, outcome measures, results, and quality analysis (Supplementary material Table 2).

### Assessment of methodological quality

The first author assessed the methodological quality of the articles using the Standard Quality Assessment Criteria for Evaluating Primary Research Papers from a Variety of Fields (Kmet et al., 2004). A second researcher also scored the methodological quality of all articles and discrepancies were resolved via discussion. The final scores are represented as a percentage indicating strong (>80%), good (70%–80%), moderate (50%–70%), or limited (<50%) methodological quality.

### Collating, summarising, and reporting the results

Included articles were summarised in relation to participant characteristics, methodology, results, or conclusions. Thematic analysis following the approach as outlined by Braun and Clarke (2022) was conducted examining themes across the findings of the included research. The framework provides a stepwise guide for collating data: (1) Becoming familiar with the data; (2) Generating initial codes; (3) Collating codes into themes; (4) Reviewing themes; (5) Defining and naming themes; and (6) Producing the report.

The first author (S.R.) developed the initial codes, which were discussed with the second author (B.A.) and were refined where necessary. The coding framework also accounted for the perspective of the research, where first order was from the experience of a participant (e.g. adolescent, parent, mentor), second order was from the perspective of the author, and third order was the interpretation of the reviewer. Within these orders, coded themes were developed for intervention components, assessment properties, outcomes, feasibility, and general feedback on the practices.

### Community involvement

Consultation with stakeholders is an optional step in the scoping review framework (Arksey & O’Malley, 2005), but was considered important in the context of this review, given increasing awareness of the need to co-produce research with autistic individuals and the autistic community (Cooperative Research Centre for Living with Autism, 2016). A reference group, consisting of five autistic young adults from varying backgrounds, was included in the analysis phase of this review. The group met twice to first discuss the study design and development of the research questions and second to discuss the findings. Group members were monetarily compensated for their time attending each meeting. Contributing to the interpretation of the data, members of the group were able to question and/or validate the findings presented by the first author.

## Results

**Figure 1.**
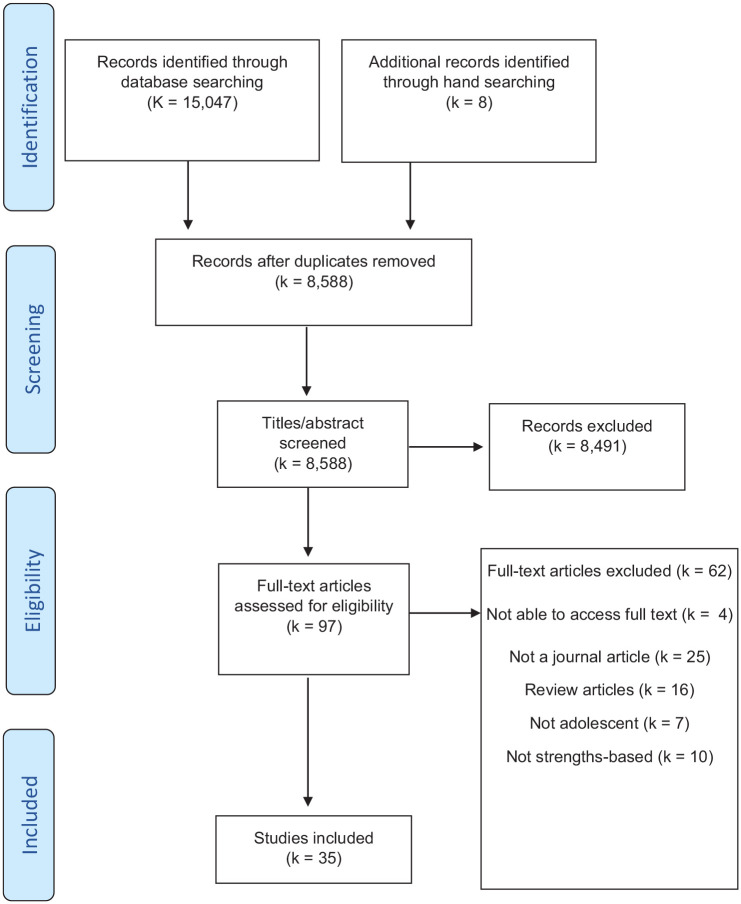
Flow chart of study selection process.

A total of 15,407 articles were identified in the initial searches, with an additional eight articles found through hand searching and reference mining. The total number was reduced to 8588 following removal of duplicates (Figure 1). Titles and abstracts were reviewed and screened according to the inclusion criteria. After abstract screening, 97 articles were retrieved for full-text screening. A final 35 articles met the inclusion criteria and were included in the review. The majority of articles were from the United States (k = 24), followed by Australia (k = 7), Canada (k = 3), and Ireland (k = 1). The studies were categorised as either articles evaluating transition-focused interventions (k = 26), or general articles relating to transition outcomes (k = 9).

### Study design and quality assessment

#### Articles evaluating transition-focused interventions (k = 26)

The methodological quality of the intervention studies ranged from limited (k = 1), to moderate (k = 4), good (k = 9), and strong (k = 12) according to the assessment developed by Kmet et al. (2004). The methodological quality of all qualitative studies (k = 7) was assessed as strong, although some partial ratings were given for the reflexivity of account, sampling strategy, and reporting of systematic data collection methods. Quantitative studies (k = 9) had notable shortcomings regarding justifying the choice of comparison group, describing subject characteristics, and reporting controlling for the impact of confounding variables. Mixed-method studies (k = 10) were analysed against the criteria of both qualitative and quantitative studies. Qualitative limitations included the sampling strategies and description of systematic data analyses, while quantitative shortcomings in mixed-method studies were similar to that of those articles employing quantitative methods only.

#### General articles relating to transition outcomes (k = 9)

The results included qualitative studies (k = 4), mixed-method studies (k = 2), articles outlining practice guidelines (k = 2), and one quantitative study. The methodological quality of the general articles ranged from moderate (k = 1), to good (k = 2), to strong (k = 4), according to the assessment developed by Kmet et al. (2004). The two guideline articles were not able to be assessed against the Kmet et al. (2004) quality guidelines.

### Active ingredients

The core components of each intervention, as attributed by the authors to produce the desired outcomes, were coded as active ingredients. For example, the technology programme evaluated by Lee, et (2020) included flexible activity options designed to ‘engage the participants in shared interests to build friendships, self-determination and skills’. The coded components were then themed and clustered. Analysis of the combinations of the themes revealed five styles of intervention: Transition Planning (k = 8), Employment Preparation and Training (k = 7), Technology Programmes (k = 4), Peer Mentoring (k = 3), and Cognitive Skills Training (k = 3). A summary of the active components of each intervention style is provided in Table 2.

**Table 2.** Description of the active ingredients for each style, as identified by clustering themes, of strengths-based interventions focused on improving transition outcomes for autistic youth.

Style of intervention	Active ingredients
Transition planning	• Career exploration • Soft skill development • Setting and reviewing transition goals • Autism awareness training
Employment preparation and training	• Development of a vocational profile (including goals, skills, and interests) • Interview training and/or work experience • Personalised feedback relating to interview or work performance • Social skills workshops
Technology programmes	• Mentoring from technical specialists • Flexible activities/project options based on interests • Promotion of social interactions though common interests • Project presentations to the group.
Peer mentoring	• Mentees and mentors matched by interests • Collaborative work on projects • Games and role-play to practice social skills
Cognitive skills training	• Workshops to increase awareness of personal strengths and challenges • Goal setting and life planning sessions • Games and role-play to practice social skills and problem-solving

### Assessment tools

Outcome measures of the intervention studies were primarily reflection-based, assessing autistic adolescents self-report of aspects of work readiness, self-efficacy, and self-determination. The majority of quantitative evaluations (k = 10) used outcome tools developed to assess specific intervention targets. However, established outcome measures included the AIR self-determination scale (k = 3) (Wolman et al., 1994), the ARC self-determination scale (k = 3) (Wehmeyer, 1995), and Goal Attainment Scaling (k = 2) (GAS; Kiresuk et al., 1994). An increase in perceived strengths was only measured as an outcome by one study (Wittevrongel et al., 2022), which concurrently assessed the acceptability of a modified version of the Ansell–Casey Life Skills Assessment (ACLSA; Nollan et al., 2000). Two additional strengths measures were utilised in ways other than assessing outcomes: the Assessment Scale for Positive Character Traits-Developmental Disabilities (ASPeCT-DD; Woodard, 2009) used to describe parent-reported strengths by Carter et al. (2015), and the Secondary School Success Checklist (SSSC; Regan et al., 2016) analysing different perspectives of transition skills by Hume et al. (2018).

### Outcomes

The strengths-related outcomes of all included intervention articles are outlined in Table 3. In qualitative and mixed-method studies examining the outcomes of transition-focused interventions, outcomes were coded as growth in specific traits according to different respondents. Those frequently identified by the participating autistic adolescents included self-advocacy, self-efficacy, social skills, work-related skills, and a sense of belonging. Those identified by caregivers included self-determination, independence, self-efficacy, social skills, self-advocacy, and self-awareness. Other informers, such as employers, mentors, and programme facilitators, mentioned outcomes in social skills, self-advocacy, confidence, and sense of belonging. Meanwhile, the predominant outcome noted by the researchers was social skills, followed by friendship, participation, self-efficacy, work-related skills, and goal setting.

**Table 3.** The strength-related intervention outcomes of transition-focused programmes for autistic adolescents as identified by different respondents.

	Outcomes:											
Reference	Self-efficacy	Friendship	Goal setting	Independence	Participation	Self-advocacy	Self-awareness	Self-determination	Sense of belonging	Social skills	Well-being	Work skills
Athamanah & Cushing (2019)				•						•		
Curtin et al. (2016)	•		•		•■					•►■	■	
DaWalt et al. (2018)	■			■		•■	■		•			
Dean, Hagiwara, et al. (2022)	►							►■				
Diener et al. (2016)		•	•		•					•		
Dunn et al. (2015)	•	•			•			•		•		•
Fullerton & Coyne (1999)	►■		•■	■		■	•►■	■	►■	■		
Goodman et al. (2017)	■	■				►					•	
Hagner et al. (2012)			•			•	•	•■				
Hatfield et al. (2017a)	•►		•				•	•				•
Hatfield, Falkmer, et al. (2018)	►■						•►					■
Hotez et al. (2018)	►					•►◆			►◆	►◆	◆	►
Jones et al. (2021)		•►						■				•►
Kaboski et al. (2015)											•	
Lee et al. (2019)	►■◆							►		■◆		•►■
Lee et al. (2020)	•	•			•■					•	•	■
Lynas (2014)	•■				►				►			•
L. A. Ruble et al. (2018)			•									
L. Ruble et al. (2019)								•			•	•
Smith et al. (2021)	•					•	•					•
Strickland et al. (2013)												•
Sung et al. (2019)	•►		•		•►		•		•	•►		•►
Wehman et al. (2013)				•						•		•
S. W. White et al. (2021)	•											
Wilson et al. (2018)	•	►			•►			•		•		
Wittevrongel et al. (2022)	■					•		■				►

Identifier code: • Researcher ► Adolescent ■ Caregiver ◆ Employer/mentor.

### Strengths

Researcher’s definitions of ‘strengths-based’ in the articles evaluating interventions most commonly referred to leveraging interests or skills. However, intervention frameworks also commonly prioritised tailored goals and activities, promoting learning rather than reinforcement, and providing opportunities to develop self-determination or self-efficacy. A range of strengths were identified as primary and secondary outcomes, as well as through feedback and observations from the researchers, participating adolescents, their caregivers, teachers, and mentors. These strengths were coded into themes and are outlined in Table 4, alongside the perspective of the respondents describing them.

**Table 4.** The strengths of autistic adolescents identified by different respondents in intervention-based and general articles relating to transition outcomes.

	**Strengths:**																											
**Reference**	Attention to detail	Caring	Confident	Creative	Empathic	Good memory	Hard working	Helpful	Honest	Humour	Imaginative	Intelligent	Kind	Loyal	Moral	Polite	Positive	Presentable	Realistic	Reliable	Resilient	Self-determined	Special interests	Systematic	Technical abilities	Thoughtful	Trustworthy	Unique
Bross & Travers (2017)																							•		•			
Carter et al. (2015)		■				•	• ■	■		•		•	• ■	■		■	•				•	•				• ■		
Diener et al. (2016)	•									•														•	•			
Hatfield et al. (2017a)																				•								
Hatfield, Ciccarelli, et al. (2018)																							• ■					
Hatfield, Falkmer, et al. (2018)						•														•								
Hotez et al. (2018)	•				•►		•		•►										►				•►	•		•►		•►
Hume et al. (2018)								■				■	►			►		•► ■		•► ■								
Kaboski et al. (2015)																							•		•			
Lee et al. (2020)				■		■						■	■	■	■								■		■		■	
Teti et al. (2016)	►	•	•►	►							►						►				•		• ►		• ►			
Wehman et al. (2017)							•													•								

Identifier code: • Researcher ► Adolescent ■ Caregiver/teacher.

The definitions of strengths in the general articles relating to transition outcomes were also coded and revealed an overarching theme of interests and skills which evoke pride. The common concepts underlying the described SB approaches included instilling a sense of purpose, developing self-advocacy, being afforded opportunities to experience success, having independence, and being in control. Furthermore, specific autistic adolescent strengths and respondents identifying these strengths across the included articles were coded into categories and are presented in Table 4.

## Discussion

This scoping review utilised thematic analysis in collating information regarding SB transition interventions and outcomes of autistic adolescents. In order to map the literature surrounding the SB approach to improving transition outcomes, two main concepts were analysed: the active ingredients of interventions and the properties of assessment tools.

### Active ingredients

A thematic review of those articles evaluating transition interventions revealed that, based on their active ingredients, transition interventions could be categorised into five types of interventions: Transition Planning, Employment Preparation and Training, Technology Programmes, Peer Mentoring, and Cognitive Skills Training.

Transition Planning programmes were centred around developing job skills, utilising goal setting and problem-solving strategies (Dean, Hagiwara, et al., 2022; Hagner et al., 2012; Hatfield et al., 2017a; L. A. Hatfield, Falkmer, et al., 2018; L. Ruble et al., 2019; Ruble et al., 2018; S. W. White et al., 2021; Wilson et al., 2018). The active components of these interventions included career exploration, soft skill development, setting and reviewing transition goals, and autism awareness information with the goal of encouraging self-advocacy. Feedback obtained in evaluating the efficacy of these programmes highlighted the importance of including the adolescent’s family in the transition planning process, particularly during goal setting and future planning (Dean, Hagiwara, et al., 2022; Hatfield, Falkmer, et al., 2018; L. Ruble et al., 2019). A qualitative examination of stakeholders’ perspectives on transition planning by Hatfield, Ciccarelli, et al. (2018) reported that 87% of parents believed they should be a key member of the transition planning team.

Employment Preparation and Training interventions incorporated work placements and programmes teaching specific job skills, such as ICT-related skills, with the aim of preparing autistic adolescents for post-school employment (Lee et al., 2019; Lynas, 2014; Smith et al., 2021; Strickland et al., 2013; Sung et al., 2019; Wehman et al., 2013; Wittevrongel et al., 2022). The active ingredients of these programmes included interview training, developing a vocational profile (including skills and interests), goal setting, providing personalised feedback on interview or work performances, and social skills workshops. The preparedness of industry partners, in terms of both their autism awareness and willingness to nurture autistic youth, was key to the success of these programmes (Lynas, 2014). One study provided autism awareness workshops for the host organisations, however, were still met with feedback that hosts would have appreciated a more in-depth understanding of the students’ capabilities and interests prior to the work placement starting (Lee et al., 2019).

Technology-based interventions were commonly delivered as extra-curricular programmes, developing autistic youth’s job skills through their interests in science, technology, engineering, arts, and mathematics (Diener et al., 2016; Dunn et al., 2015; Jones et al., 2021; Lee et al., 2020). Some of the reviewed programmes were run as intensive weeklong workshops (Diener et al., 2016; Dunn et al., 2015), while others were delivered as weekly sessions during the school year (Jones et al., 2021). These programmes were characterised by similar active components, regardless of their delivery format, focusing on mentoring from technical specialists, delivering flexible activities and project options, promoting of social interactions between peers in various ways, and presenting projects to their peers.

Interventions centred around Peer Mentoring included mentoring for incoming university students, peer supported work-based learning, and paired extra-curricular programmes (Athamanah & Cushing, 2019; Curtin et al., 2016; Hotez et al., 2018; Kaboski et al., 2015). Active components included engaging in games and role-play to practice social skills, goal setting and planning, and collaborative work on projects. Matching mentors and mentees by interests was recognised by each study as integral for the success of the programme. Some studies also reported positive outcomes in self-advocacy for mentees and mentors alike (Hotez et al., 2018).

Finally, Cognitive Skills Training programmes focused on fostering character traits likely to improve work readiness, including confidence, self-determination, and self-advocacy (DaWalt et al., 2018; Fullerton & Coyne, 1999; Goodman et al., 2017). Active components included information sessions to increase awareness of the targeted traits, goal setting and life planning sessions, and group activities such as role-playing social situations. One study focused on delivering cognitive training to families as a whole (DaWalt et al., 2018), reporting that the active ingredients of the programme was effective in improving both autistic adolescents and their parents confidence in their problem-solving abilities.

### Assessment tools

While goal setting was highlighted across the included articles as an important strategy in delivering transition programmes, only two articles measured autistic adolescents’ goal attainment (Dean, Hagiwara, et al., 2022; L. A. Ruble et al., 2018). Both of these studies employed Goal Attainment Scaling (GAS; L. Ruble et al., 2012), a process involving establishing intervention goals and identifying outcomes indicating progress towards those goals. Given the individualised nature of GAS, establishing the validity and reliability of this measure is difficult, a limitation addressed by training of facilitators in developing scales which are measurable, equidistant, and unidirectional (Dean, Hagiwara, et al., 2022). One study described using a rigorous Psychometrically Equivalence Tested Goal Attainment Scaling (PET-GAS; L. Ruble et al., 2012), incorporating a number of procedures across multiple raters to ensure high quality, comparable goals, and attainment scales. A content analysis of adolescents’ transition GAS goals by Dean, Burke, et al. (2022) revealed adolescents establish goals across five categories: enhancing self-management; obtaining employment; exploring career opportunities; enhancing learning; and enhancing self-advocacy. These categories could potentially be used as a framework for guiding the development of GAS goals in future SB programme evaluations.

Self-determination is defined as the interaction between capacity and opportunity, being positively correlated with quality of life (Shogren et al., 2015; K. White et al., 2018), and positive transition outcomes (Shogren et al., 2008b). Studies in the present review measured this outcome using either the AIR self-determination scale (Hatfield, et al., 2017a; S. W. White et al., 2021; Wolman et al., 1994) or the ARC self-determination scale (Dean, Hagiwara, et al., 2022; Hagner et al., 2012; Wehmeyer, 1995) to assess changes following a transition programme. While both scales have been reported to have strong psychometric properties, a study comparing the measures reported the ARC scale was more sensitive in measuring changes in intra-individual characteristics, constructs likely to be more relevant in assessing changes in transition-based knowledge and behaviour (Shogren et al., 2008a).

Only three explicit strength measures were identified in the reviewed articles, two of which may be used to assess tangible skills in the development of a transition plan. The first of these was the Secondary School Success Checklist (SSSC), which collects the perspectives of students, their parents, and their teachers on skills learnt in secondary school which translate to various domains in adulthood (Hume et al., 2018). The respondent rates the student’s level of a skill (e.g. ‘I bring everything I need to my classes’), from ‘not like me’ to ‘very much Like me’, and rate their priority to learn that skill, from ‘I would not like to learn this’ to ‘I really want to learn this’, which supports the individualisation of the tool in transition planning. The measure has been found to have moderate to high internal consistency across informants; however, the authors reported higher agreement between the perspectives of teachers and parents, compared with agreement between adolescents and either group (Hume et al., 2018).

The second measure of tangible skills was the Ansell–Casey Life Skills Assessment (ACLSA; Nollan et al., 2000), used to measure employment readiness, occupational focus and action, and well-being. The original measure has been reported to have adequate internal consistency and reliability in neurotypical populations but has not been evaluated with a neurodivergent sample (Nollan et al., 2000; Wittevrongel et al., 2022). The study by Wittevrongel et al. (2022) adapted a modified version (ACLSA-M) that focused on life skills essential for employment for autistic individuals, including job readiness, autism-related traits (e.g. social skills), and well-being. The ACLSA-M has not been psychometrically evaluated with a neurodivergent sample and, although it met the pre-determined level of acceptability, stakeholders had several recommendations to improve the relevance of the measure (Wittevrongel et al., 2022).

Between the two, the SSSC may be more suitable for autistic populations, as it was developed through an iterative process that considered their unique strengths and challenges, and accounts for individual priorities (Hume et al., 2018). However, the ACLSA-M emphasises independence in areas such as daily living, housing and money management, and self-care, without accounting for individual priorities. Although these skills are important for transition, some items may not be as relevant or achievable for all autistic individuals and the measure has the potential to marginalise those who thrive in supportive environments or who benefit from collaborative approaches (Boele, 2017; Sosnowy et al., 2018). The need for co-produced refinement of the ACLSA-M is evident in the findings of Wittevrongel et al. (2022), outlining items in the domains of relationships and communication, work and study life, self-care, and career and life planning, which should be included to accurately represent independence of autistic students.

The third strengths measure identified was the Assessment Scale for Positive Character Traits-Developmental Disabilities (ASPeCT-DD), which explores parents’ perspectives of their adolescents’ strengths (Carter et al., 2015). The ASPeCT-DD differs from the SSSC and ACLSA in that it is a measure of strengths – innate qualities that shape character (Niemiec et al., 2017) – rather than learnt skills. For example, the measure assesses the qualities of Courage (e.g. *I think my child is courageous*), Empathy (e.g. *My child shows caring for other people*), and Forgiveness (e.g. *My child does not try to retaliate or get back at others who have hurt him or her*). The research by Carter et al. (2015) revealed that all adolescents had at least one strength and that individual strength-profiles varied widely. However, the measure is not specific to autism and may not adequately capture traits and strengths that are particularly relevant to autistic individuals, such as specific social communication skills or unique problem-solving abilities (Russell et al., 2019). This could inadvertently cause the measure to focus more on challenges or deficits, rather than highlighting the positive attributes and strengths of autistic participants (Niemiec et al., 2017).

Overall, no established measure of character strengths specific for autistic adolescents was identified. This gap can lead to the underrepresentation of unique talents, as autistic strengths may be overlooked in educational and workplace settings, thereby missing opportunities to cultivate abilities that could enhance personal growth and development (Scott et al., 2018). Recent advances to include strengths assessment in the diagnosis process has led to the development of the Survey of Autistic Strengths, Skills, and Interests (SSASI; Woods & Estes, 2023), designed to allow clinicians to assess strengths commonly associated with autism in both adults and children. Where the SSSC may be the most appropriate measure for assessing transition skills and setting goals, an autism-specific character strengths measure, such as the SSASI, has the potential to improve the individualised approach to transition and increase individual’s confidence and self-esteem (Kapp et al., 2013; Lee et al., 2020; Russell et al., 2019). This sentiment was echoed by the reference group of this review, none of whom could recall completing a measure that helped them in understanding their personal strengths. The group highlighted the importance of an adaptable measure, with one member suggesting the use of open-ended questions and/or the space to provide more information if the participant felt the answer was too nuanced for a dichotomous or Likert-type scale response.

### Outcomes

Analysis of the intervention articles revealed 13 SB outcomes; the most common being increases in self-efficacy, identified in 19 out of the 26 articles. Self-efficacy is defined as one’s belief in his or her capability to successfully perform a particular task and is considered a key driver of autonomy (Bandura, 1977; Littlewood, 1996). Self-determination and self-advocacy were also common outcomes of the interventions, identified in nine and six of the articles, respectively. These concepts have collectively been defined as the ability to take initiative over one’s life and accept the consequences of one’s actions (Test et al., 2020, 2009). Research has shown that all of these concepts are strong predictors of success in both employment and post-secondary education for autistic youth (Test et al., 2020), providing empirical support for these SB outcomes as intervention targets.

The second most prominent outcome in the present review was Work Skills, which covered a range of employment readiness skills including interviewing, networking, and portfolio development. The importance of this SB outcome has been supported by Test et al. (2020) through evidence that the development of students work attitudes and behaviours equips adolescents for greater success. Similarly, career-specific preparation and development of technical skills has been identified as a predictor for post-secondary success (Mazzotti et al., 2021; Test et al., 2020). This was also captured by the Work Skills outcomes in the present review, which included the ICT-related skills that participants developed in the Technology Programmes.

Some of the outcomes directly stem from the core components of the interventions, such as goal setting and social skills. For example, several interventions that focused on helping individuals identify, plan, and evaluate personal or vocational goals also measured goal setting or attainment as an outcome. Similarly, social skills training was identified as a key element in many of the interventions, with 10 of the studies reporting improvements in social skills as an outcome. Both goal setting and social skills are well established as predictors of success in post-secondary education and employment (Mazzotti et al., 2016; Test et al., 2020). Therefore, their inclusion as both outcomes and active components of the interventions is strongly supported by evidence. However, the inclusion of social skills training in the interventions was flagged by the autistic reference group of this study as a potential issue in SB approaches and is explored further under the next section.

A number of the present outcomes have not been supported empirically, including friendship, sense of belonging, and well-being. This may in part be due to the measurement of these outcomes being qualitative, meaning they are subjective by nature, whereas evidence-based outcomes tend to be robust, quantitative measures (Guldberg, 2017; Odom et al., 2010).

### Implications for practice

In discussing the findings with the reference group of autistic young adults, concerns were raised regarding the inclusion of social skills training as an active ingredient in a number of the SB interventions, questioning whether these practices truly adopted a strength’s perspective. The concern is that traditional social skills training may inadvertently promote compliance with neurotypical norms, leading to masking which can negatively impact mental health (Cassidy et al., 2019; Hull et al., 2018). Approaches to social skills training which maintain a strengths base should begin with consulting the individual about their goals, be flexible and adaptable to the participants feedback, promote authenticity, and utilise inclusive language and practices (Afsharnejad et al., 2022; Donaldson et al., 2017; Monahan et al., 2023).

Similarly, it was highlighted that cognitive skills training interventions should be tailored towards individual needs in ensuring that strategies were generalised beyond the programmes, to participants everyday lives. The group suggested prioritisation of individual assessments and goals, the incorporation of holistic supports that acknowledge challenges as well as strengths, and the provision of real-world practice opportunities to enhance the generalisation of skills learned. Furthermore, participants expressed the importance that programmes actively involve autistic individuals in the design and evaluation processes. Overall, these insights advocate for a person-centred, flexible approach in designing transition programmes that genuinely align with individual needs and fostering authentic self-expression rather than conformity.

### Research gaps and future directions

This scoping review has revealed a major gap in the measurement of strengths in autistic adolescent populations, with most studies failing to use a standardised outcome measure, instead employing measures designed specifically for the study unsupported by robust psychometric evaluation. The absence of a standardised strengths measure should be addressed through the development and/or validation of a scale for explicit strengths appraisal in autistic adolescents. Such a scale could be used as an outcome measure as well as a tool in self-discovery.

Additionally, while a number of the SB outcomes identified in this review have been supported by empirical evidence, some are backed by emerging evidence requiring further investigation and others are not endorsed at all in the literature (Mazzotti et al., 2016; Test et al., 2009). This warrants further validation of the SB outcomes and their link to post-secondary outcomes for autistic adolescents. Empirical evidence of the outcomes as post-secondary successes would also support the development of measurement frameworks.

Given the importance of providing a person-centred approach (Hatfield, Ciccarelli, et al., 2018; Thompson et al., 2018), further research should be conducted to compare and contrast the priorities of different stakeholders. Such research would be beneficial in the development of interventions going forward to ensure they are meeting the needs and expectations of the autistic community. These results also support the need for coproduction and including autistic youth in the research processes which concern their outcomes. Engaging autistic youth throughout the development of an intervention will allow for their voice to be heard at multiple stages, rather than just through feedback and revision (Cooperative Research Centre for Living with Autism, 2016).

### Limitations

A primary limitation of this scoping review is the emerging nature of SB research and practices which lends to the subjective definition of strengths. While great effort was made to include texts with broad and ranging definitions, there may be relevant articles that were excluded due to an ambiguous definition of strengths. In a similar vein, a large number of studies were found to include autistic participants under a broader umbrella of disability. While these may have provided further insights to SB transition outcomes, they were omitted due to not being specific to autism, as was the objective of this review. Finally, the inclusion criteria of articles published in English yielded a large proportion of research from the United States and could be considered an unequal representation of SB transition outcomes across countries.

### Conclusion

This scoping review has demonstrated the variabilities and similarities of SB approaches to improving transition outcomes for autistic adolescents. While there is an emerging body of work examining SB approaches in autism and our understanding is evolving, there is still the need for more work to be done until we can completely define what SB approaches are for autistic adolescents. However, through the classification of intervention types and description of SB transition outcomes, this review can be used to inform future frameworks. This review has highlighted the importance of leveraging each individual’s interests and skills, developing individualised processes, and accommodating participants needs through a holistic approach. This research will assist future SB programmes to align with the priorities of autistic adolescents and their parents.

## Supplemental Material

sj-pdf-1-aut-10.1177_13623613251346336 – Supplemental material for A scoping review of current approaches to strengths-based transition practices for autistic adolescents Supplemental material, sj-pdf-1-aut-10.1177_13623613251346336 for A scoping review of current approaches to strengths-based transition practices for autistic adolescents by Sophie Rumsa, Bahareh Afsharnejad, Elinda Ai Lim Lee, Sven Bölte, Tele Tan and Sonya Girdler in Autism
