# Supplementary material for: A scoping review of current approaches to strengths-based transition practices for autistic adolescents
Source: Autism. 2025 Jun 25;29(11):2644–61. doi: 10.1177/13623613251346336 (PMC12531391; doi:10.1177/13623613251346336)
Supplement: sj-pdf-1-aut-10.1177_13623613251346336 – Supplemental material for A scoping review of current approaches to strengths-based transition practices for autistic adolescents [file sj-pdf-1-aut-10.1177_13623613251346336.pdf]

Author (year) country	Sample	Aim	Method	Duration	Intervention	Outcome measures	Results	Quality
Athamanah and Cushing (2019) USA	N = 6<br>14-18 years.<br>n = 3 with ASD	Assess the impact of a peer-mediated intervention on the vocational skills and social interactions between students with ASD and their peers in an integrated work-based learning setting in high school.	Pre-test/ post-test	16 weeks	**(M)** Peer mediated work-based learning in which mentors were taught research-based strategies of modelling, prompting, and scaffolding to support their peer in the workplace	Study specific recording system of independent engagement in vocational tasks, social interactions, and the quality of the social interactions	Increased independence in vocational tasks, social interactions, and improved the quality of social interactions for all three dyads.	Moderate
Curtin et al. (2016) USA	N = 9<br>13 – 18 years	Evaluate the feasibility of providing one-to-one mentoring for adolescents with ASD based in a community recreation program setting.	Pre-test, post-test and follow up measures	6 months (once weekly)	**(M)** In the Partners Exploring Education and Recreation (PEER) mentoring program, mentors and mentees choose from a menu of activities, including arts, education, leadership, life skills, sports, fitness and recreation, and technology	1. Paediatric Quality of Life Scale<br>2. Rosenberg Self-Esteem Scale<br>3. Social Worries Questionnaire<br>4. Youth Survey: Measuring the Quality of Mentor-Youth Relationship<br>5. Study specific satisfaction questionnaire	Improved participants' social connectedness	Moderate
DaWalt et al. (2018) USA	N = 90<br>n = 45 adolesc 14 – 17 years<br>n = 45 parents	Evaluation of a multi-family group psychoeducation intervention	Pre-test/ Post-test randomis ed control trial (waitlist control)	8 weeks	**(CT)** Transitioning Together intervention involves 2 individual family joining sessions, 8 weekly parent and teen group sessions, and ongoing resources and referrals. The adolescent social group involved learning activities and	1. Centre for Epidemiological Studies Depression scale<br>2. Perceived Stress Scale<br>3. Family Empowerment Scale<br>4. Positive Affect Index<br>5. Zarit Burden Interview<br>7. National Longitudinal Study of Adolescent Health	Significant improvements in parental depressive symptoms and problem solving for parents, and improvements in social interactions for youth in the intervention condition relative to	Strong

Table 1. Descriptive characteristics of intervention based articles. Intervention type: M: Mentoring, CT: Cognitive training, TP: Transition planning, T: Technology, ET: Employment training

Author (year) country	Sample	Aim	Method	Duration	Intervention	Outcome measures	Results	Quality
					games on topics such as sharing interests, goal setting, problem solving, and social planning.	8. Social Responsiveness Scale<br>9. Study specific questionnaires and observational ratings.	controls. Parents reported satisfaction with the program and particularly valued the opportunity to interact with other families.	
Dean et al. (2022) USA	N = 22<br>14-23 years	Investigate the feasibility and preliminary impact of the Self-Determined Career Design Model (SDCDM) with youth and young adults with ASD	Quasi-experimental pre-/post-test	Varied duration of 1-hour weekly sessions	**(TP)** The SDCDM consists of a three-phase problem solving process which are further divided into Person Questions, Facilitator Objectives, and Employment Supports. The program is implemented by a trained facilitator to supports the person's career-related goals	1. Canadian Occupational Performance Measure<br>2. Goal Attainment Scaling<br>3. The Arc's Self-Determination Scale<br>4. survey of items previously used in the disability employment literature	Significant gains in goal attainment and occupational performance. Participants found aspects of the SDCDM useful, particularly enhancing skills related to self-determination.	Strong
Diener et al. (2016) USA	N = 7<br>8-17 years<br>All male	Explore the processes occurring during technology workshops which build on interests and enhanced social engagement	Qualitative thematic analysis	7 weeks (one week intensive then weekly)	**(T)** Students developed a 3D design using online software SketchUp with group instructions by a certified instructor and mentor, who teaches tool use and demonstrates the software features.	N/A	The two key themes:<br>1. development of peer relationships through humour, common interests, physical actions, and playful competition; and<br>2. the importance of a scaffolded learning environment with support from peers	Strong

Table 1. (Continued)

Author (year) country	Sample	Aim	Method	Duration	Intervention	Outcome measures	Results	Quality
							and an adult mentor, as opportunities for social engagement.	
Dunn et al. (2015), USA	N = 8<br>9-19 years<br>All male	Explore engagement and learning in a technology-based extracurricular program	Grounded theory analysis	5 days (3 hours per day)	**(T)** The iSTAR program is an extracurricular technology program for youth with ASD that employs graphic design software (SketchUp) as a way to promote social engagement and to explore technology as a potential vocation	N/A	*Youth Centred Learning* and *Opportunities to Demonstrate Skills* emerged as themes that illuminated the processes by which engagement and learning occurred.	Good
Fullerton and Coyne (1999), USA	N = 46<br>n = 23 adolesc 16-23 years<br>n = 23 parents	(a) Explore the impact of specific instructional methods and activities for assisting young adults with autism to develop the awareness and the skills needed for self-determination, (b) identify the challenges students face in understanding and applying concepts related to self-determination, and (c) examine to what extent young adults have applied concepts	Survey & pre- / post-interviews	10 sessions (2-3 hours)	**(CT)** Seven units consisting of visual and written information, as well as group activities, enabling students to gain the necessary social understanding, self-awareness, and self-directed actions needed for self-determination.	N/A	Students indicated that the instructional strategies used had been helpful. They may benefit from an opportunity to share experiences with people their own age who have similar strengths and challenges. The students used communication skills taught in the class in other settings. A majority of the students were more	Strong

Table 1. (Continued)

Author (year) country	Sample	Aim	Method	Duration	Intervention	Outcome measures	Results	Quality
		and strategies in their own lives.					able to plan the steps needed to accomplish their goals in the interview context and at home initiated more discussion of their goals. Some students (37%) also engaged in more self-directed actions toward goals.	
Goodman et al. (2017), Canada	N = 8<br>14 -17 years	Undertake the preliminary work required to develop a metacognitive training (MCT) program for high-functioning adolescents with ASD. Observing and documenting the strengths and limitations of the MCT protocol.	Mixed feasibility	7 x 90-minute sessions	**(CT)** Group training sessions included lectures, interactive activities and discussions, covering topics such as executive functioning, mental flexibility, and big-picture thinking. participants worked towards a common goal - the development of a presentation on cognitive differences in ASD and personal strengths and challenges to share with their parents.	1. Piers–Harris Children's Self-Concept Scale Second Edition<br>2.Children's Depression Inventory – Second Edition	Self-esteem did not decrease during or after MCT nor did depressive symptoms increase.	Good
Hagner et al. (2012), USA	N = 94<br>n = 47 students	Assess the effectiveness of a family centred	Randomiz ed control trial	1 school year	**(TP)** Family centred transition process empowers students with ASD and their	1. Adaptive Behavior Assessment Scale–II<br>2. Autism Diagnostic Observation Schedule	The experimental group reported significantly higher student expectations	Good

Table 1. (Continued)

Author (year) country	Sample	Aim	Method	Duration	Intervention	Outcome measures	Results	Quality
	16 - 19 years<br>n = 47 parents	transition planning approach	(waitlist control)		families, educates them about the transition process, and helps them connect with community resources on the transition readiness of youth with ASD.	3. The expectations section of the survey used for the National Longitudinal Transition Study–2<br>4. Arc Self-Determination Scale (Adolescent Version)<br>5. Vocational Decision-Making Interview–Revised questionnaires	for the future, parent expectations for the future, self-determination, and vocational decision-making ability. None of these variables improved significantly for control group.	
Hatfield et al. (2017), Australia	N = 94<br>13 - 17 years<br>(45 in the control group)	Determine the effectiveness of the BOOST-A in improving self-determination among adolescents on the autism spectrum.	Quasi-randomised controlled trial.	12 months	**(TP)** The Better OutcOmes & Successful Transitions for Autism (BOOST-A): An online transition planning program which utilises self-awareness and goal setting	1. The Social Responsiveness Scale–Second Edition<br>2. AIR Self-Determination Scale<br>3. the Career Development Inventory—Australia—Short Form<br>4. Personal Wellbeing Index- School Children<br>5. Learning Climate Questionnaire<br>6. Study specific questionnaire	Most outcomes improved over time, with greater improvements for the intervention group. Significant differences in favour of the intervention group in three areas: opportunity for self-determination at home as reported by parents; career exploration as reported by parents and adolescents; and transition-specific self-determination as reported by parents.	Strong
Hatfield, Falkmer, et al.	N = 72<br>n = 33 adolesc	Identify and describe the effectiveness, usability, facilitators,	Process evaluation	4 x 1 hour sessions	**(TP)** The Better OutcOmes & Successful Transitions	Study specific questionnaire relating to the BOOST-A intervention	Quantitative: 82% of parents/67% of adolescents agreed	Strong

Table 1. (Continued)

Author (year) country	Sample	Aim	Method	Duration	Intervention	Outcome measures	Results	Quality
(2018), Australia	12 – 17 years n = 39 parents	and barriers of the BOOST-A transition planning program			for Autism (BOOST-A): An online transition planning program which utilises self-awareness and goal setting		that the BOOST-A™ was easy to use. 57% of parents/ 49% of adolescents agreed that the BOOST-A™ helped their child/them to prepare for leaving school and 90% of parents/46% of adolescents agreed that they would recommend the program to another person. Qualitative: four core themes: (i) taking action to overcome inertia, (ii) new insights that led to clear plans for the future, (iii) adolescent empowerment through strengths focus, and (iv) having a champion to guide the way.	
Hotez et al. (2018), USA	N = 23 17 – 28 years	Evaluate the program's impact on self-advocacy, academic self-efficacy and self-reported ASD symptoms. Determine	Quasi-experimental pre-/post-test	One week	**(M)** Weekly individualized one-on-one and/or group mentorship sessions with a structured curriculum. Workshops	1. The Social Responsiveness Scale 2. Disability Identity Scale 3. Academic Self-Efficacy Scale	Increase in ASD knowledge and a decrease in self-reported ASD symptoms was observed from pre-	Good

Table 1. (Continued)

Author (year) country	Sample	Aim	Method	Duration	Intervention	Outcome measures	Results	Quality
		recommendations for future programs.			introduce a skill, demonstrate the appropriate use of the skill, and then ask each student to use the skill with a peer.	4. An adapted version of the Autism Awareness Survey<br>5. Test of Nonverbal Intelligence	to post-test. No changes in disability identity or academic self-efficacy were observed.	
Jones et al. (2021), Australia	N = 68<br>n = 23 adolesc 10-18 years<br>n = 25 parents<br>n = 20 facilitator s	Identify the essential components of strength-based technology clubs by exploring context, mechanisms, and outcomes of existing strength-based technology clubs	Ethnograp hic methods	Various lengths (1 - 10 weeks)	**(T)** Three interventions specialising in technology or computer coding: Autism Academy of Software Quality Assurance (AASQA) CoderDojo, Autism West, and Firetech Australia.	N/A	Four context themes: *personal factors of adolescents, personal factors of facilitators, personal factors of parents, institution*<br>Three mechanism themes: *activity design, strengths and abilities, environment*<br>Three outcome themes: *skill building, connection with others, emotion*	Strong
Kaboski et al. (2015), USA	N = 16<br>12 – 17 years<br>n = 8 with ASD	Evaluate a novel intervention designed to reduce social anxiety and improve social/ vocational skills for adolescents with autism spectrum disorder	Pre-test/post-test	5 days (3-hour sessions)	**(M)** A weeklong pilot summer robotics camp during which participants learned robotic facts, actively programmed an interactive robot, and learned career skills	1. Social Anxiety Scale for Children-Revised (SASC- R) or Social Anxiety Scale Adolescents (SAS-A) depending on their age.<br>2. Social Skills Improvement System<br>3. Study specific questionnaire	The ASD group showed a significant reduction in self-reported social anxiety but no statistically significant improvement in social skills.	Moderate
Lee et al. (2019), Australia	N = 17<br>n = 5 adolesc	Explore the key factors contributing to successful work placement experience	Grounded theory approach	5–10-day work placeme nts	**(ET)** Work placement in an ICT related organisation to provide an early	N/A	Key factors contributing to success included preparing for the	Strong

Table 1. (Continued)

Author (year) country	Sample	Aim	Method	Duration	Intervention	Outcome measures	Results	Quality
	15 - 18 years n = 6 parents n = 6 employers	and the perceived benefits of these placements			exposure to a real workplace that matches the interests, strengths, skills, and abilities of the adolescents with ASD.		workplace, harnessing strengths and interests, and developing work related skills, while the benefits include insight into the workplace, recognising potential, working as a team and the pathway ahead	
Lee et al. (2020), Australia	N = 52 Parents of autistic adolesc	Explore the principal components contributing to the success of a community-based strengths-based program and the outcomes for autistic students as a result of participating in the program	Longitudinal survey	10 weeks (2 hours/week) and 1 year (2-3 hours/week)	**(T)** The Autism Academy of Software Quality Assurance (AASQA) CoderDojo program allows autistic students to develop their special interests in Science, Technology, Engineering, Arts and Mathematics, through mentors supporting them to work on digital projects, learn and practice computer programming.	1. Autism Spectrum Quotient (AQ): Adolescent Version 2. Study specific questionnaires and scales	Approximately 60% reported improvements in the ICF chapters relating to communication (63%), interpersonal interactions and relationships (61%) and major life areas (58%). 40% either agreed or strongly agreed their child had improved in the component of community, social and civil life.	Strong
Lynas (2014), Ireland	N = 72 n = 42 students 16< years n = 30 adults.	Evaluate the success of Project ABLE in its aim to support young people and adults with learning difficulties or ASD to secure	Action research approach	Varied per participant	**(ET)** Peer mediated work-based learning (job shadowing, paid and unpaid work experiences, internships, school-	N/A	56% of the adult group achieved full time and part time employment in a variety of sectors. Feedback from	Limited

Table 1. (Continued)

Author (year) country	Sample	Aim	Method	Duration	Intervention	Outcome measures	Results	Quality
		employment and achieve greater independence			based work activities situated either in the school or in a community workplace)		participants highlighted significant benefits in social skills, independence, confidence, communication, anxiety, behaviour, vocational skills, physical health. 97% of participants have developed or improved their vocational, social and communication skills and consequently have become more independent in various aspects of their lives.	
Ruble et al. (2018), USA	N = 60<br>n = 20 students 17 – 20 years<br>n = 20 teachers<br>n = 20 parents	Evaluate the feasibility of the Collaborative Model for Promoting Competence and Success (COMPASS) for transition planning and implementation.	Randomised control trial	6 months of a session every 5 weeks	**(TP)** Collaborative Model for Promoting Competence and Success (COMPASS): Consultation and coaching to achieve personal transition goals	1. Vineland Adaptive Behavior Scales, Second Edition (VABS II)<br>2. Behavior Assessment System for Children, Second Edition (BASC-2)<br>3. Childhood Autism Rating Scale, Second Edition<br>4. Study specific checklists assessing consultant and teacher adherence<br>6. Psychometrically Equivalence Tested Goal Attainment Scaling	67% of students who received COMPASS met their stated goal compared to only 18% of those in the control group. High consultant fidelity was rated by parent, teacher, and independent research ratings. Teaching fidelity varied across time; adherence was	Moderate

Table 1. (Continued)

Author (year) country	Sample	Aim	Method	Duration	Intervention	Outcome measures	Results	Quality
							lowest for the early coaching sessions and improved significantly over time.	
Ruble et al. (2019), USA	N = 40<br>n = 4 ASD individ.<br>n = 11 parents<br>n = 8 school providers<br>n = 6 policy makers<br>n = 3 adult service providers<br>n = 10 autism committe e	Understand the barriers and facilitators of transition planning and implementation for students with ASD	Mixed-method explorator y research	10< hours	**(TP)** Collaborative Model for Promoting Competence and Success (COMPASS): Consultation and coaching to achieve personal transition goals	Study specific questionnaires and scales	Five factors impacting outcomes were identified: a need for a coordinator, a need to account for the heterogeneity of autism, need for addressing problems that youth with ASD face when obtaining employment, needed practical skills in current transition implementation, need to look beyond employment as the primary transition outcome. Teachers strongly agreed that the modified version of COMPASS was acceptable and usable.	Good
Smith et al. (2021), USA	N = 71<br>16 – 26 years	Evaluate the initial feasibility and effectiveness of VIT-TAY among autistic	Randomis ed control trial	15 sessions (45 minutes)	**(ET)** Virtual Interview Training aims to improve job interview skills, job interview	1.Brief version of the Mood and Feelings Questionnaire (b-MFQ)	The intervention group showed significant improvements in	Good

Table 1. (Continued)

Author (year) country	Sample	Aim	Method	Duration	Intervention	Outcome measures	Results	Quality
		transition aged youth when implemented by teachers in high school settings			self-efficacy, job interview anxiety, and access to employment.	2. National Institutes of Health (NIH) Toolbox Cognition Battery<br>3. standardized child behaviour checklist (CBCL) or adult behaviour checklist (ABCL)<br>4. Adapted Treatment Acceptability Rating Form<br>5. Mock Interview Rating Scale adapted for autism (A-MIRS).<br>6. Brief Personal Report of Public Speaking Apprehension (PRSPA)<br>8. study specific questionnaires	overall job interview skills and reduced job interview anxiety. Improvements were largely in confidence, positively framing experiences, demonstrating professionalism, sharing strengths and skills, and sharing limitations.	
Strickland et al. (2013), USA.	N = 22<br>16 – 19 years<br>All male	Evaluate the effectiveness of a treatment package comprised of a web-based interviewing skills program (JobTIPS) and virtual reality practice on responses to employment interview questions	Randomised control trial	1 week	**(ET)** JobTIPS: a web-based interviewing skills program and virtual reality practice on responses to employment interview questions	1. Interview Skills Rating Instrument with two sub-scales: Response Content and Response Delivery<br>2. Social Responsiveness Scale	The intervention group showed a significant positive change at the second interview on the Content Scale and a trend toward a positive change on the Delivery Scale. The program was more effective in teaching ‘‘content’’ rather than ‘‘delivery’’ skills.	Strong

Table 1. (Continued)

Author (year) country	Sample	Aim	Method	Duration	Intervention	Outcome measures	Results	Quality
Sung et al. (2019), USA	N = 17<br>18 – 25 years	Assess feasibility and preliminary efficacy of the Assistive Soft Skills and Employment Training (ASSET) program in promoting self-perceived soft skills and self-efficacy.	Quasi-experimental pre-/post-test	8 weeks (90 minutes a week)	**(ET)** The ASSET program covers six skills: communication, enthusiasm and attitude, teamwork, networking, problem-solving and critical thinking, and professionalism**.** Delivered in a group format to create opportunities to explicitly practice skills through peer interaction with the guidance of group facilitators and provision of immediate feedback	1. Social Responsiveness Scale<br>2. Wechsler Abbreviated Scale of Intelligence–Second Edition<br>3. The Perceived Empathy Self-Efficacy Scale (PESE)<br>4. The Perceived Social Self-Efficacy Scale (PSSE)<br>5. Study-specific questionnaires	Significant improvements in work-related social skills knowledge, social functioning, and self-efficacy. High satisfaction with program activities, training modalities, and delivery of the intervention.	Good
Wehman et al. (2013), USA	N = 2<br>19 & 20 years<br>Both male	Present the components of Project SEARCH and how they were adapted for youth with ASD in a model program, describe the problem-solving approach we used when implementing Project SEARCH for youth with ASD, and examine two autistics students and their internships.	Case study	3 x 10 - 12-week internships	**(ET)** Project SEARCH: a high school program that provides a unique school-to-work transition model through combining real-life work experience, training in employability and independent living skills, and placement assistance. Students spend their entire school day at the workplace for a full	Study specific marking rubric covering seven dimensions of job skills and behaviours:<br>1.performance of job skills<br>2. overall production rate<br>3. accuracy<br>4. communication<br>5. interaction with coworkers<br>6. appearance<br>7. safety	Participant 1 made progress throughout all areas in the internships. The data also demonstrated that his independence in his second and third internships increased along with his social behaviour.<br>Participant 2 had consistent social behaviour and job performance across	Good

Table 1. (Continued)

Author (year) country	Sample	Aim	Method	Duration	Intervention	Outcome measures	Results	Quality
					school year to facilitate a seamless integration.		the first and second internships, however, his social behaviour and job performance evaluation rose significantly, with the addition of behavioural supports in the third internship.	
White et al. (2021), USA	N = 59<br>16 – 25 years	Evaluate the feasibility and preliminary efficacy of the Stepped Transition in Education Program (STEPS) for Students with ASD.	Randomised control trial	16 weeks (1 hour per week)	**(TP)** STEP 1 for secondary students involved counselling and activities to prepare for postsecondary education	1. Achenbach System of Empirically Based Assessment (ASEBA)<br>2. American Institutes for Research Self-Determination Scale (AIR-SD)<br>3. Social Responsiveness Scale–Second Edition<br>4. Student Adaptation to College Questionnaire (SACQ)<br>5. Transition Readiness Scale (TRS)<br>6. Wechsler Abbreviated Scale of Intelligence, Second Edition<br>7. Study specific Satisfaction Survey	STEP 1: The baseline model indicated a significant immediate positive treatment effect. The long-term effect suggested that treatment effects did not diminish over time, though there was a tendency for transition readiness to decline toward baseline.<br>STEP 2: The results of the baseline model suggested a significant and positive immediate treatment effect.	Strong
Wilson et al. (2018), Canada	N = 10<br>15 - 21 years<br>All male	Examine the perspectives of adolescents with ASD during their	Deductive thematic analysis	4 weeks	**(TP)** Participants set goals then were guided on how to develop concept maps	N/A	Five themes emerged:	Strong

Table 1. (Continued)

Author (year) country	Sample	Aim	Method	Duration	Intervention	Outcome measures	Results	Quality
		participation in a novel occupationally based intervention			using software. The global problem-solving framework of CO-OP (Goal, Plan, Do, Check) was used to support the participants as they expanded on their maps and individualized their plans to meet their unique needs.		1. finding a sense of balance through negotiating tensions 2. a sense of "we" and a sense of "I 3. selecting purposeful, meaningful, and authentic occupations 4. multimodal tools 5. action through participating in doing.	
Wittevrongel et al. (2022), Canada	N = 187 n = 177 adolesc 15 – 29 years n = 10 researche rs	Evaluate the acceptability of the Work Readiness Inventory (WRI) and the Ansell–Casey Life Skills Assessment (ACLSA) as measures of employment readiness in youth and young adults on the spectrum.	Concurren t triangulati on mixed-method design	undefine d	**(ET)** Employment readiness program focused on social, communication, and skill development to improve employment readiness, support occupation selection, and provide work exposure. Achieved through mentorship-based training and workplace Exposure.	The Work Readiness Inventory Ansell–Casey Life Skills Assessment	Significant change in employment readiness was evident in both scales after participants completed an employment readiness program. Both measures were deemed acceptable in assessing employment readiness in youth and young adults on the spectrum.	Good

Table 1. (Continued)

Author (year), country	Sample	Aim	Methodology	Outcome measures	Results	Quality
Bross and Travers (2017), USA	N/A	Outline a process for providing school-based opportunities which align with a student's special interest areas	Expert opinion using a case example to outline the process	N/A	The process includes 4 steps:<br>1. conducting preference assessments to identify SIAs,<br>2. matching school-based opportunities to the SIA,<br>3. developing a plan to embed instruction, and<br>4. evaluating SIA intervention effects.	N/A
Carter et al. (2015), USA	N = 427 caregivers of adolescents (13 – 21 yrs) with autism (42.9%) or an intellectual disability	Identify the strengths of youth and young adults with intellectual and developmental disabilities from the vantage point of their parents	Mixed-method exploratory research using closed-ended measures and open-ended interviews.	Assessment Scale for Positive Character Traits–Developmental Disabilities (Aspects-DD)	Every young person in the sample was described as having at least one strength (Mdn = 20, range 1-26), and their strength-related profiles varied widely. Challenging behaviours predicted lower ratings.	Good
Dean et al. (2022), USA	N = 21<br>(14 – 23 yrs)	Explore the career design goals set by autistic youth engaged in the self-determined career design model (SDCDM) intervention	Qualitative content analysis of career goals	Goal Attainment Scaling (GAS)	21 goals, organised into five categories: enhancing self-management, obtaining employment, exploring career opportunities, enhancing learning, and self-advocacy	Good
Hatfield, Ciccarelli, et al. (2018), Australia	N = 162<br>n = 26 adolescents (13 – 17 yrs)<br>n = 83 parents<br>n = 53 professionals	Needs assessment to determine the predisposing, reinforcing, and enabling factors that impact on successful transition	Mixed-method exploratory research using a questionnaire with both closed- and open-ended questions.	Study specific online questionnaire.	65% reported they would have liked transition planning to start earlier. Predisposing, reinforcing, and enabling factors are described.	Strong

Table 2. Descriptive characteristics of general articles relating to transition outcomes.

Author (year), country	Sample	Aim	Methodology	Outcome measures	Results	Quality
Hume et al. (2018), USA	N = unclear<br>n = 539 adolescents (13 – 20 yrs)<br>n = 541 parents<br>n = unclear teachers	Determine level of transition-related skills as reported by adolescents, their parents, and their teachers. Describe the perceived importance of each skill and examine relationships between the perceptions of skill proficiencies across informant groups	Quantitative exploratory research using mean comparisons of the responses from different informant groups on the same questionnaire.	Secondary School Success Checklist (SSSC)	All three informant groups similarly identified four of the highest rated skills. There was no agreement across all three groups when identifying the lowest rated skills.	Moderate
Roberts (2010), USA	N/A	Provide educators and transition support personnel with topics to consider when working with students with ASD and their families to develop a transition plan	Expert opinion based upon existing literature regarding transition processes.	N/A	Topic areas include career exploration, academic goal setting and preparation, assessing and knowing learning styles, self-advocacy skills, accommodations, academic supports, interagency collaboration, technology, and time management.	N/A
Sosnowy et al. (2018), USA	N = 41<br>n = 20 young adults (18 – 29 yrs)<br>n = 21 parents of autistic young adults	Collected the perspectives of parents of young adults with ASD and young adults with ASD in order to understand how they view desired outcomes, barriers, and facilitators, and identify strategies, services, and supports that may help the transition to adulthood.	Qualitative exploratory research using a grounded theory approach to analyse semi-structured interviews	N/A	Parents and young adults view postsecondary outcomes as complex and integrated concepts rather than discrete, disconnected categories. Living independently, postsecondary education, and work were seen as means to achieving broader goals, not just measurable achievements.	Strong
Teti et al. (2016), USA	N = 11<br>(16 – 22 yrs)	Investigate which experiences young adults with ASD describe as having the most influence on them as they move into adulthood.	Qualitative exploratory research using Photovoice for young adults to express their experiences of growing up with ASD	N/A	Three sub-themes describe youth's strengths: 1) special interests that cultivated positive emotions and coping strategies; 2) skills and activities that evoked pride; and 3) reframing ASD as special versus a disadvantage.	Strong

Table 2. (Continued)

Author (year), country	Sample	Aim	Methodology	Outcome measures	Results	Quality
Thompson et al. (2018), Australia	N = 19 parents of autistic young adults (18 – 30 yrs)	Explore the viewpoints of parents of young adults with ASD in relation to their child's transition to adulthood.	Qualitative exploratory research using an inductive approach to analyse the outcomes of four structured focus group discussions	N/A	Three major themes emerged: to be understood, to understand the world and to succeed. The ICF domains of activity and participation and environmental factors emerged as having the greatest potential to influence transition outcomes	Strong

Table 2. (Continued)
